# Investigating preliminary usability of a novel self‐administered cognitive assessment on an iPad

**DOI:** 10.1002/alz70857_105029

**Published:** 2025-12-24

**Authors:** Ravneet K. Dhaliwal, Jacklyn Gehling, Hannah Marx, Steven Harris, Najeeb Qadi, Bushra Siddiqui, Claudia Jacova

**Affiliations:** ^1^ Pacific University, Hillsboro, OR, USA; ^2^ Cogni.Dx, London, England, United Kingdom

## Abstract

**Background:**

Traditional cognitive assessments for older adults can be time‐consuming, resource‐intensive, and often inaccessible in primary care. The integration of electronic devices into assessment procedures could improve efficiency and access. We investigated the preliminary usability of a novel iPad‐based assessment tool for online self‐administration in an older adult sample with differing technology proficiency.

**Method:**

Participants were 20 community‐dwelling older adults, free of neurocognitive diagnoses, aged 64‐88 (M=74.15) 65% female, 90% White, with an average of 16.25 years of education. They completed the Mobile Device Proficiency Questionnaire (MDPQ‐16), range 24 to 80 (M=58.35), indicating a wide range of proficiencies. They were provided an iPad and an instructions packet to self‐administer the cognitive assessment in office with supervision (*n* = 10) or at home with no supervision (*n* = 10). The cognitive assessment (developed with Cogni.Dx) consisted of five tests probing orientation, visuospatial memory, language, and visuomotor speed (trails). Usability was evaluated by thematically analyzing and categorizing researcher observations, participant requests for help, and participant feedback (oral and written) on difficulties encountered during setup and completion.

**Result:**

Nineteen participants were able to complete the cognitive assessment (9: home, 10: office). Various usability issues emerged that are categorized into four themes: set‐up, interruptions, touchscreen interface, and extraneous concerns. In the home condition, participants had trouble activating or logging into their account and connecting the device to their home WiFi. Three more difficulties were observed in both home and office conditions. One was interrupting the testing despite instructions to complete it in a single sitting. Another challenge was the use of the touchscreen interface, particularly for tests requiring moving shapes or drawing lines where calibrating pressure of touch was observed to be very challenging. Lastly, extraneous concerns, such as pop‐ups, scrolling to see the entire page, closing out of the module by mistake, and re‐navigating to the assessment were also challenges.

**Conclusion:**

Preliminary findings revealed multiple usability challenges encountered by participants in both home and office conditions. These challenges of set‐up, interruptions, touchscreen interface navigation, and managing extraneous technical issues, underscore the importance of thorough usability investigations before considering validity and widespread implementation.

